# Why are tapes better than wires in knotless rotator cuff repairs? An evaluation of force, pressure and contact area in a tendon bone unit mechanical model

**DOI:** 10.1186/s40634-020-00321-y

**Published:** 2021-02-03

**Authors:** Carlos Maia Dias, Sérgio B. Gonçalves, António Completo, Martina Tognini, Manuel Ribeiro da Silva, Jorge Mineiro, Francisco Curate, Frederico Ferreira, João Folgado

**Affiliations:** 1 Department of Bioengineering, and iBB - Institute for Bioengineering and Biosciences, Instituto Superior Técnico, Universidade de Lisboa, Lisbon, Portugal; 2 IDMEC, Instituto Superior Técnico, Universidade de Lisboa, Lisbon, Portugal; 3 TEMA, Department of Mechanical Engineering, University of Aveiro (UA), Aveiro, Portugal; 4 Politecnico Di Milano, Milan, Italy; 5 Hospital de São João, Porto, Portugal; 6 Hospital CUF Descobertas, Lisboa, Portugal; 7 Laboratory of Forensic Anthropology, Department of Life Sciences, University of Coimbra, Coimbra, Portugal; 8 Department of Bioengineering, and iBB - Institute for Bioengineering and Biosciences, Instituto Superior Técnico, Universidade de Lisboa, Lisbon, Portugal

**Keywords:** Rotator, Cuff, Tape, Wire, Suture, Force, Pressure, Area

## Abstract

**Purpose:**

Knotless repairs have demonstrated encouraging performance regarding retear rate reduction, but literature aiming at identifying the specific variables responsible for these results is scarce and conflictive.

The purpose of this paper was to evaluate the effect of the material (tape or wire suture) and medial tendon passage (single or double passage) on the contact force, pressure and area at the tendon bone interface in order to identify the key factors responsible for this repairs´ success.

**Methods:**

A specific knotless transosseous equivalent cuff repair was simulated using 2 tape or suture wire loaded medial anchors and 2 lateral anchors, with controlled lateral suture limb tension. The repair was performed in a previously validated sawbones® mechanical model. Testing analyzed force, pressure and area in a predetermined and constant size “repair box” using a Tekscan® sensor, as well as peak force and pressure, force applied by specific sutures and force variation along the repair box.

**Results:**

Tapes generate lower contact force and pressure and double medial passage at the medial tendon is associated with higher contact area. Suture wires generate higher peak force and pressure on the repair and higher mean force in their tendon path and at the medial bearing row. Force values decrease from medial to lateral and from posterior to anterior independently of the material or medial passage.

**Conclusion:**

Contrary to most biomechanical literature, suture tape use lowers the pressure and force applied at the tendon bone junction, while higher number of suture passage points medially increases the area of contact. These findings may explain the superior clinical results obtained with the use uf suture tapes because its smaller compressive effect over the tendon may create a better perfusion environment healing while maintaining adequate biomechanical stability.

**Supplementary Information:**

The online version contains supplementary material available at 10.1186/s40634-020-00321-y.

## Introduction

Rotator cuff tears are common and its surgical treatment is becoming increasingly frequent [13]. Repair integrity has been shown to correlate with clinical and strength improvement [5, 10, 12, 31, 36, 57] but non-healing and retear rates still remain high [1, 21].

Minimization of motion at the tendon footprint (tendon-bone interface (TBI)), its anatomical restoration, adequate initial fixation strength and low tension on the repaired tendon have demonstrated to be important factors for tendon healing [16, 41]. Aiming to reach such benefits, new repair techniques such as trans-osseous equivalent (TOE) and suture-bridge (SB) repairs were developed [10, 48, 54] and tended to overcome double and single-row repairs in terms of footprint coverage, tendon-bone contact pressure, gap formation and ultimate load to failure [8, 19, 41, 51, 55].

Tying the medial row, using Mason-Allen stitches and having multiple sutures passages in the tendon were other technical approaches that showed to contribute to an increase in the stability of the TBI at time 0 [3, 24, 32, 43, 44, 49, 56].

Stiffer and more stable constructs, such as the ones previously mentioned, helped to reduce retear rate [9, 21, 38, 49, 52], especially in large sized tears. However, a concerning shift towards type 2 retears [14](medial to the repair site)occurred [4, 10, 25, 54] as these are substantially more complex and difficult to treat.

In this context, the use of suture tapes instead of wires for knotless TOE repairs was proposed as they theoretically allowed a better distribution of compressive forces on the cuff, enhanced self-reinforcement [37, 42] and showed a smaller abrasive effect than wires [6, 15, 18, 28, 30, 53], but some authors found conflictive results [23, 31]. Most probably, more stable constructs reduce retear rates, but those that occur are more serious and difficult to treat, therefore no clear gold standard technique has been established.

Evaluating TOE and SB repairs in detail and identifying particular factors that can contribute to maintain their mechanical benefits without inducing type 2 retears seems important. Such factors may include the type of material used for the repair, the number of sutures passed in the medial cuff and allowing suture sliding in that specific region.

Literature comparing tapes and suture wires used in shoulder repairs settings is scarce and most of it is either focused on the mechanical properties of suture materials or explores its failure mechanism [15, 18, 53]. Very few studies evaluated the differences in terms of force, pressure and contact area and even fewer compared homogenous groups.

The current study aims to compare tapes and suture wires in that setting, and to the best of our knowledge, for the first time, to evaluate the mechanical consequences (namely contact force, pressure and area) at the TBI of passing one or two sutures from the medial anchors in a single hole at the medial cuff.

We hypothesized that under the same mechanical conditions, suture tapes increase force, pressure and contact area in the tendon bone junction and that suture limbs passed individually (double passage group) in the medial cuff also increase contact area.

## Material and methods

### Experimental setup

#### Measured parameters and materials used

Total contact force, pressure and area, as well as footprint loading pattern of 4-four different knotless TOE repairs were evaluated using a Tekscan® 5051 pressure mapping sensor (Tekscan Inc.®, Boston, MA). The sensor is constituted by a flexible array of 46 × 46 force sensors, presenting a spatial resolution of 62 sensors per cm^2^. To avoid damaging its surface with punctures by sutures and needles, the sensor was folded to fit the area under the tendon model. The sensor was posteriorly calibrated using a Shimadzu® calibrator (Shimadzu Corporation©, Kyoto, Japan). In order to increase the resolution of the analysis, the maximum pressure was defined to 0.69 MPA, a value 39 times higher than the normal systolic blood pressure (< 130/80 mmHg) [2]. Calibration settings were saved and reproduced in all the tests.

To ensure homogeneity between testing samples we chose to use SAWBONES® SKU 1521–12-2 training model (SAWBONES®, Vashon, WA) instead of cadaveric tissue to simulate tendon-bone interface. This type of model consists of a rigid foam that mimics the mechanical properties of the humeral head. This model also includes a neoprene foam that replaces the tendon, albeit not trying to replicate its mechanical characteristics. SAWBONES models have been previously used by the medical and biomechanics community to perform their training and research activities, being considered a valid tool for comparative analysis when the biological aspects are not relevant or when they induce experimental variability (e.g. analysis of orientation of the acetabular cup in osteotomy techniques, anchor fixation testing and rotator cuff repair evaluation) [17, 20, 48].

#### Test groups

**Fig. 1:**
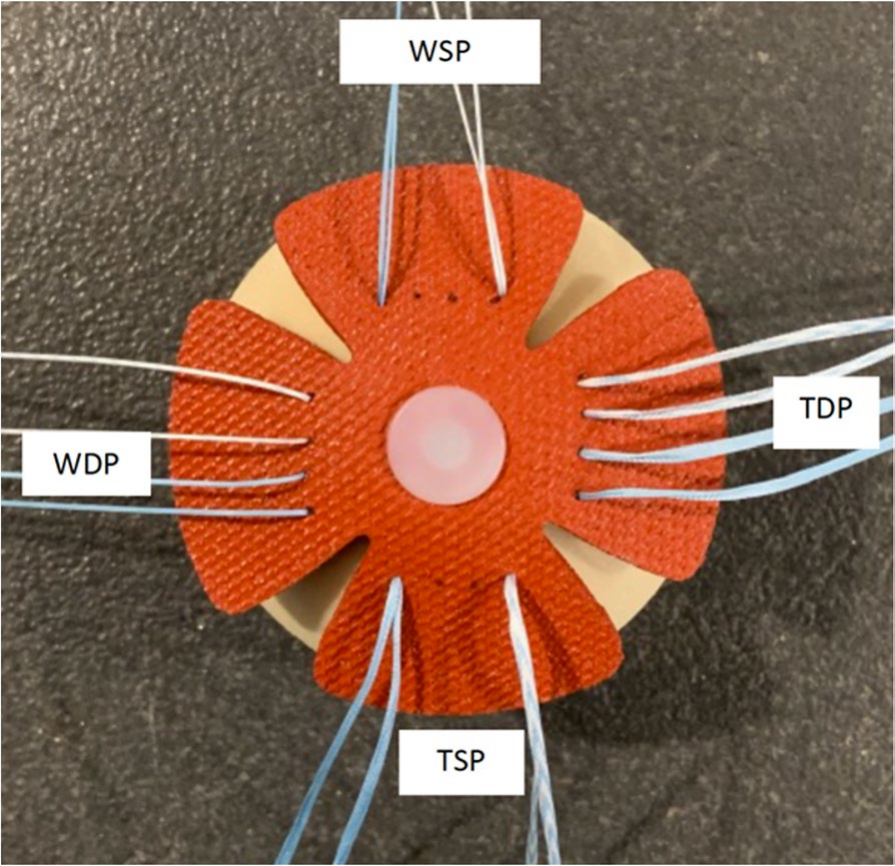
Different types of repair according to the type of suture and medial passage. TSP—Tape Single Passage; TDP -Tape Double Passage; WSP—Wire Single Passage; WDP—Wire Double Passage

Four different types of knotless TOE repairs were performed (4 test groups). The groups differed in the type of suture used (tape or suture wire) and in the type of medial passage (single passage, in which both wire or tape limbs were passed in a single hole or double passage, in which each suture/tape limb from one of the medial anchors passed individually in the simulated tendon) (see Fig. 1):

- Group 1—TSP (Tape/Single passage);
- Group 2—TDP (Tape/Double passage);
- Group 3—WSP (Wire/Single passage);
- Group 4—WDP (Wire/Double passage).

#### Mock surgical technique description

The mock repairs were performed using two Helicoil® 5.5 mm anchors (Smith & Nephew, London, UK) for the medial row, both of them either loaded with one Ultrabraid® suture (wire) or with one Ultratape® suture (tape). These anchors allow suture slide in its eyelet. For the lateral row, two 5.5 mm Footprint Ultra PK® anchors (Smith & Nephew, London, UK) were used. Five trials were repeated for each test group.

**Fig. 2:**
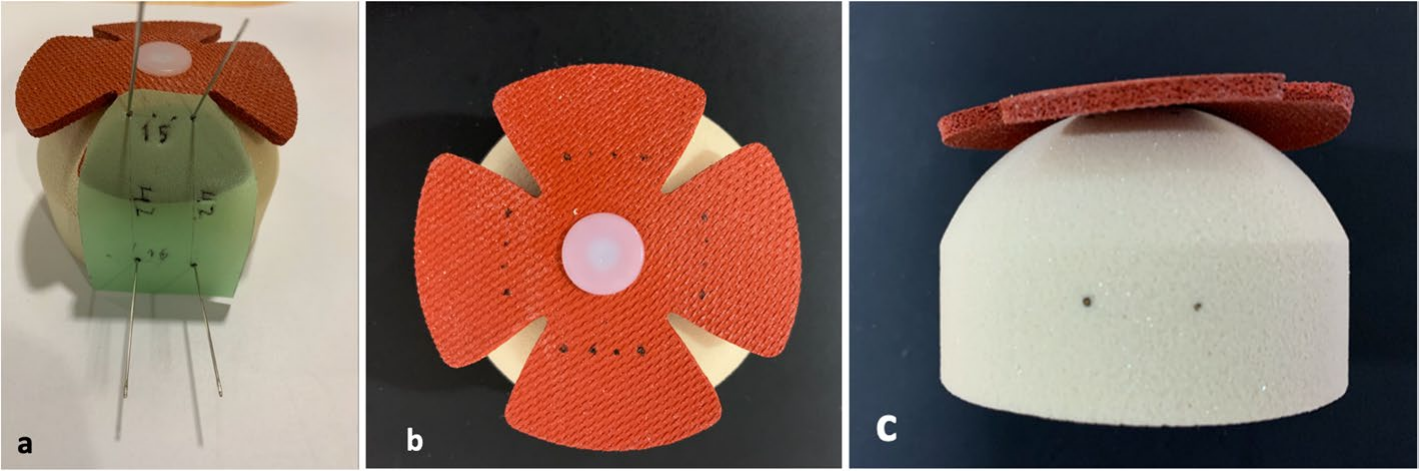
**a** Templating and medial and lateral anchor location marking with needles in the simulated bone; **b** Suture passage location markings after templating; **c** Lateral anchor location marking after templating

A flexible plastic template was used to ensure that all anchors and sutures were reproducibly placed (see Fig. 2a, b, c). Tapes and wires were passed in the mock tendon, either in a single or double passage fashion, using for that purpose the same single-sized needle in all trials. The sensor was placed under the tendon model and held with finger pressure. One suture limb (tape or wire) of each medial anchor was pulled and placed in the anterolateral (AL) anchor. The AL was always placed before the posterolateral (PL) anchor, with the sutures slacked to avoid undetermined tensioning. Sutures limbs were then individually pulled and tensioned using 2 suture tensioners (EU000715 Suture Tensioner, Smith and Nephew, London, UK®) previously calibrated, which allow measurement of four different tension values: 25, 50, 75 and 100 N. The sutures were tensioned until sliding occurred. The anchor was then locked and the tensioners released. In order to prevent backward sliding when pulling on the remaining sutures, a clamp was placed in the AL locked suture limbs.

**Fig. 3:**
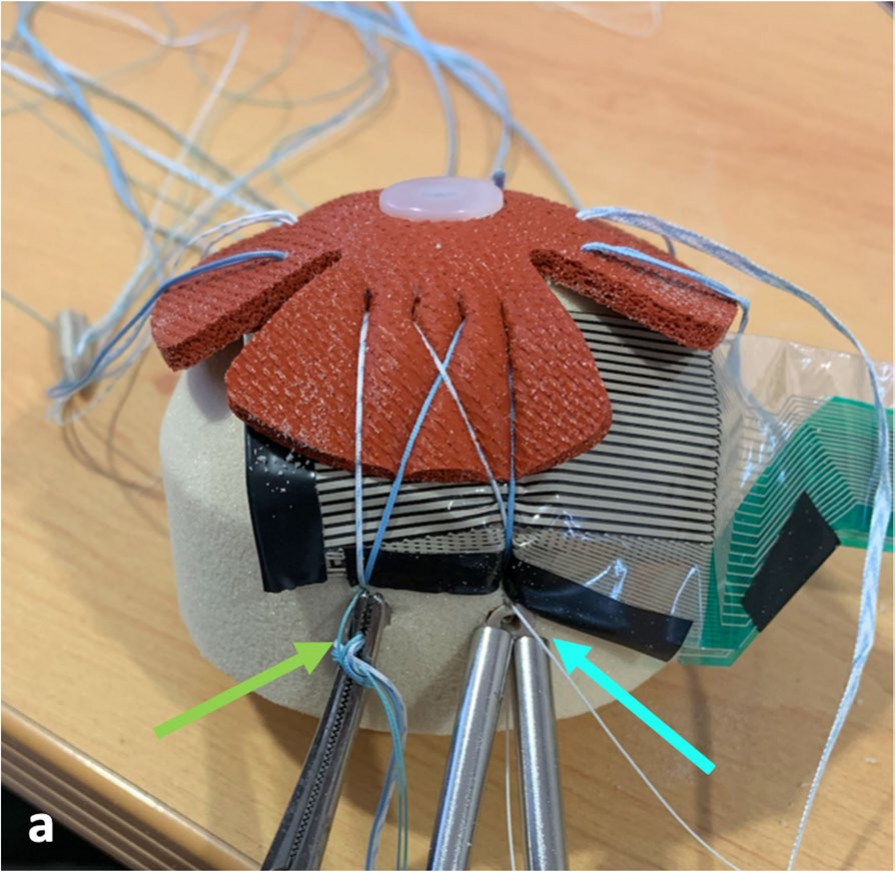
Wire Double passage (WDP) trial with clamp protecting sutures sliding from the antero lateral anchor (see green arrow) and both suture tensioners pulling suture limbs placed in the postero lateral anchor (blue arrow) with the sensor beneath the tendon

The PL anchor was then placed following the same sequential steps. In this case a tension of 75 N was applied in both suture limbs (see Fig. 3). Sensor finger stabilization was released when sufficient contact to the mechanical model allowed sensor stable positioning. At that time a mapping of force, pressure and area at the TBI was acquired using the I-Scan Lite software (Tekscan Inc.®, Boston, MA).

The assemblies were made by the same shoulder fellowship trained surgeon in order to increase trial homogeneity.

### Data analysis

The analysis of the contact force, pressure distribution and contact area were made on I-Scan Lite® software. The single cell saturation was set for 0.69 MPa, the maximum pressure applied during the calibration procedure. A repair region of 729 mm^2^ (27 × 27 mm), i.e. the “Repair Box” was defined on the acquisition software for each preparation for total force, pressure and contact area comparison. An analysis of the maximum peak force and pressure for an area of sixteen (4 × 4) force cells (25.81mm^2^) and its location was also performed.

**Fig. 4:**
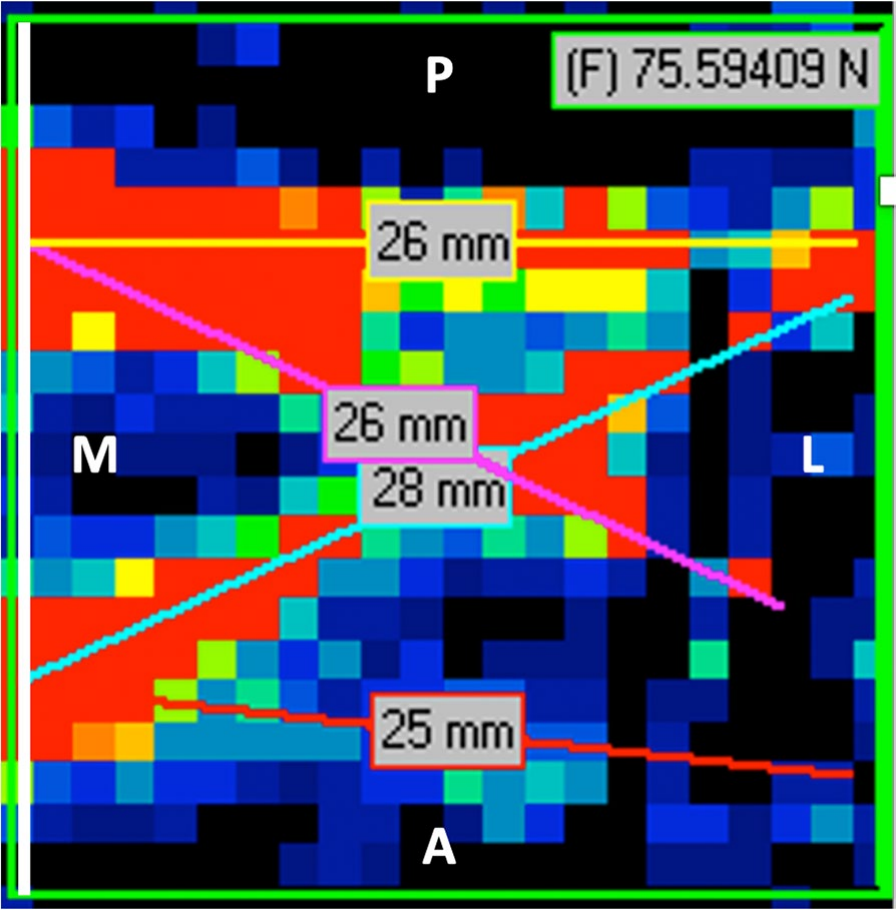
Repair box (green square) example evaluated by I-scan lite software® (A – anterior; P- Posterior; M- medial; L—lateral). Red line represents the antero medial – antero lateral suture; Pink line represents the postero medial- antero lateral suture; Blue line represents the antero medial – postero lateral suture; Yellow line represents the postero medial – postero-lateral suture; White line (most medial line of the box) represents the antero medial- postero medial line

Force distribution along the medio—lateral (Box ML) and posterior—anterior direction (Box PA) was measured to analyze its distribution pattern in the different repair types. The average force applied by the sutures in each sensor (force per sensor) was also evaluated in all trials (see Fig. 4). The four different sutures were defined according to their direction in the construct:

- AM-AL – anteromedial to anterolateral suture;
- AM-PL – anteromedial to posterolateral suture;
- PM-AL – posteromedial to anterolateral suture;
- PM-PL – posteromedial to posterolateral.

An additional AM-PM (anteromedial to posteromedial) line was established to evaluate the contact force in the medial bearing row, which is the most medial area of apposition of the tendon to the bone. In this case, the value presented was not the average force / sensor, but actually the total force along that specific line as its size was constant for every essay.

The computation of the force values per sensor in the suture path and force variation in the “Repair Box” region was performed using MATLAB software (The MathWorks, Inc., Natick, MA).

### Statistical analysis

Descriptive statistics was applied for all variables and for variance group analysis. A Kruskal–Wallis test with a null hypothesis that group results were similar were used for comparison of the different types of repairs. A post-hoc analysis with Bonferroni correction for multiple tests was also applied to infer the existence of differences between the four individual groups. For analysis of differences between tapes and suture wires and between single and double medial passage, a Mann–Whitney test was applied. The statistical analysis was performed on IBM SPSS Statistics v26 software (IBM, Armonk, NY). A level of significance of 5% was used for all the statistical analyses.

## Results

### Total contact force, area and pressure in the repair box

**Fig. 5:**
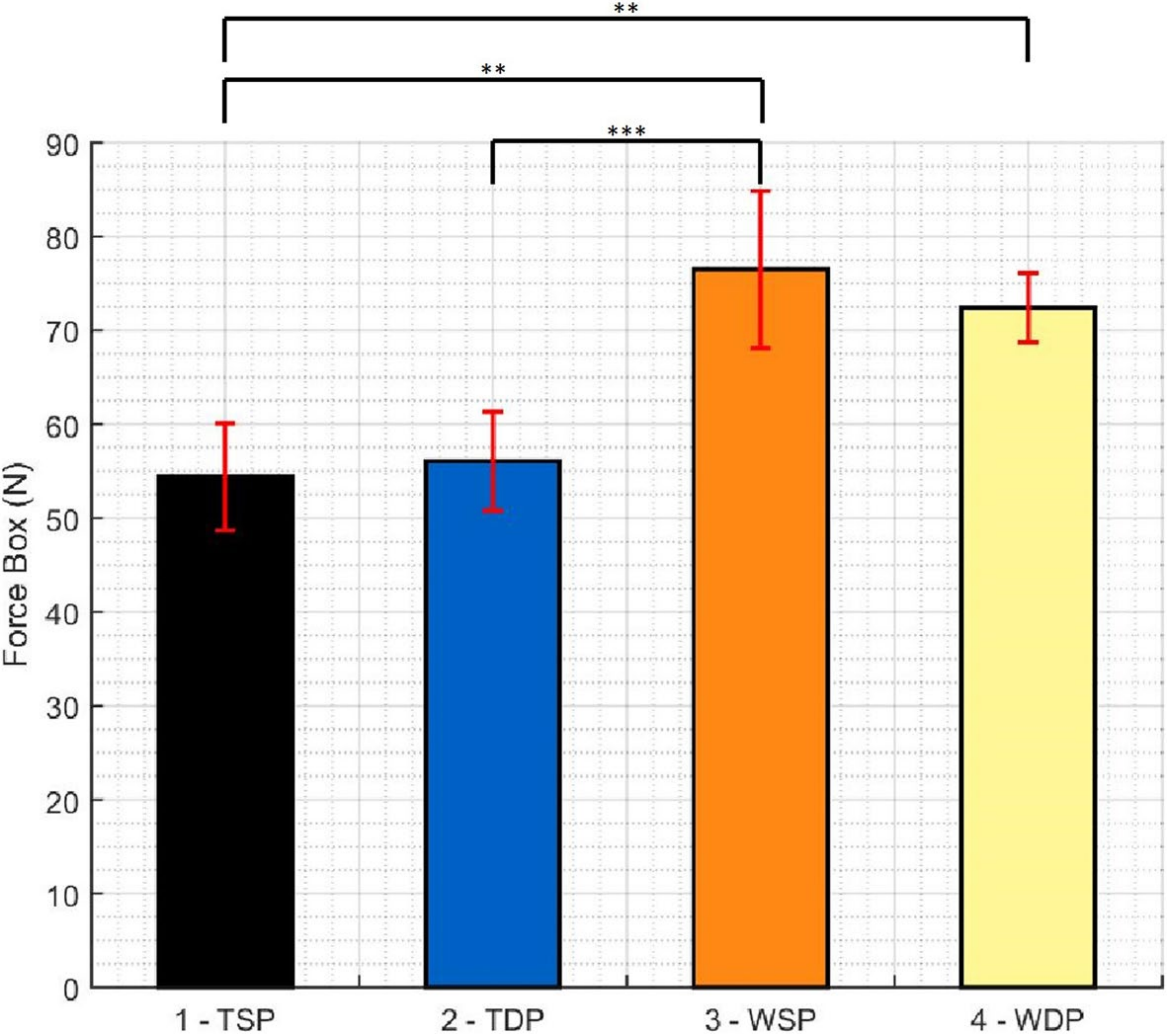
Pairwise comparison of all groups regarding total force in the repair box (* p < 0.01, ** p < 0.005, *** p < 0.001)

**Fig. 6:**
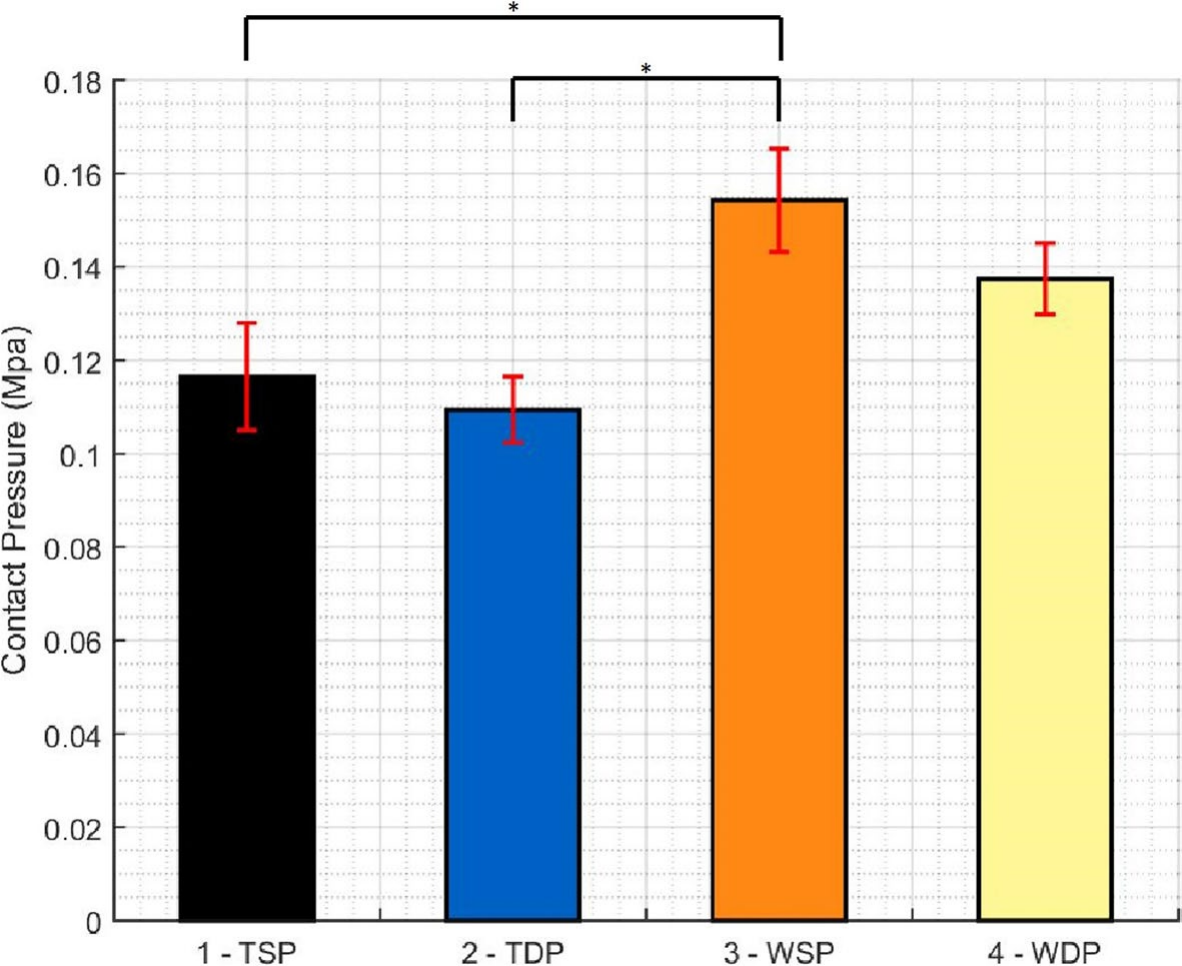
Pairwise comparison of all groups regarding total contact pressure in the repair box (* p < 0.01, ** p < 0.005, *** p < 0.001)

**Fig. 7:**
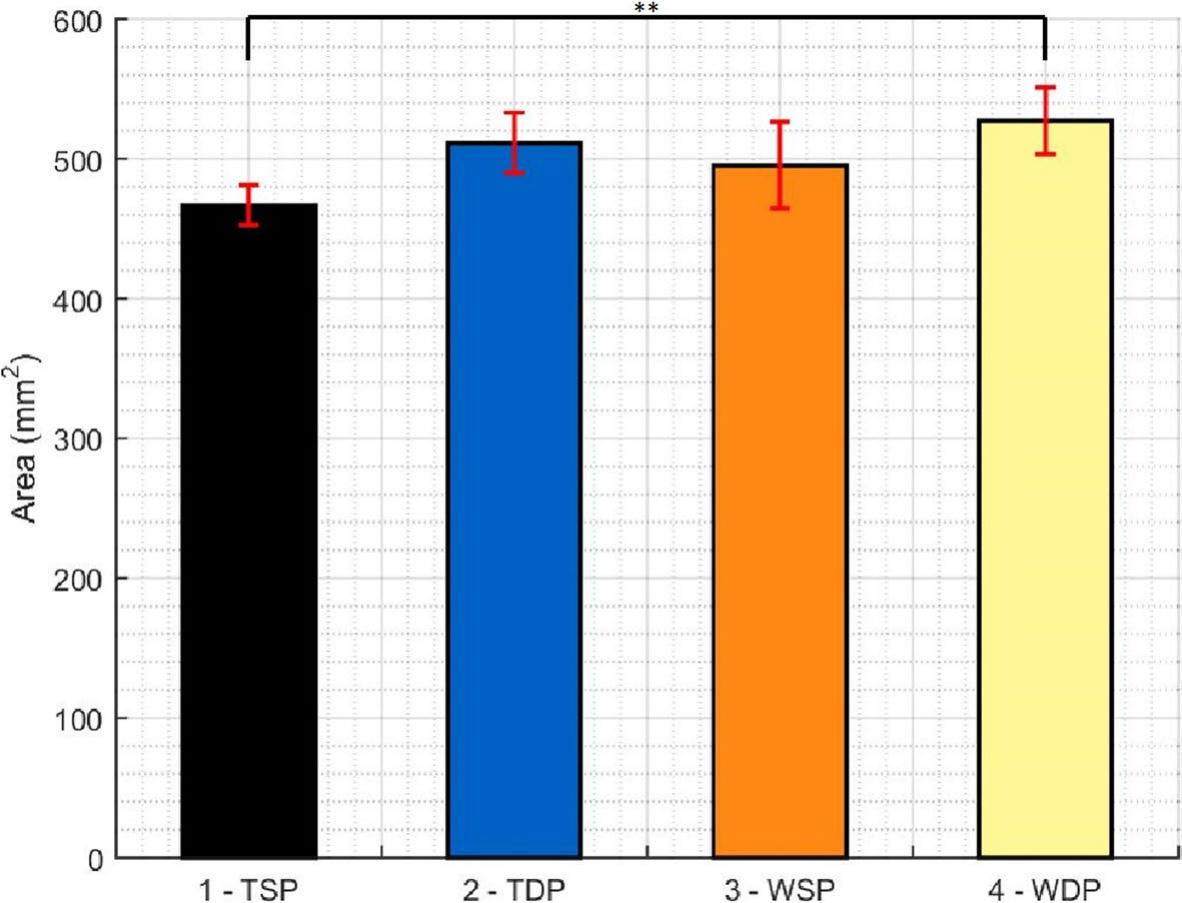
Pairwise comparison of all groups regarding total contact area in the repair box (* p < 0.01, ** p < 0.005, *** p < 0.001)

Table 1 summarizes results regarding total contact force, pressure and contact area in the “Repair Box”. While WSP presents the highest total contact force and pressure, TSP and TDP showed the lowest total contact force and pressure respectively. WDP showed the highest total contact area of all groups, at values significantly different from the lowest value, obtained by the TSP group. Figures 5, 6 and 7 show the pairwise comparisons between all groups.

**Table 1:** Descriptive Statistics (Total contact force, area and pressure)

	**TSP**	**TDP**	**WSP**	**WDP**
**Force** (N)				
**Mean**	54.38	56.04	76.49	72.44
St Dev	5.71	5.28	8.36	3.69
**Area** (mm^2^)				
**Mean**	466.80	511.40	495.40	527.40
St Dev	14.31	21.65	31.01	23.77
**Pressure** (MPa)				
**Mean**	.1165	.1094	.1542	.1375
St Dev	.01152	.00711	.01105	.00762

When comparing single and double passage groups, independent of the material used, significant differences were only found in total contact area, with higher values for medial double passage (*p* = 0.011).

When comparing tape and wire repairs disregarding the type of medial passage, wire repairs showed statistically significant higher total contact force and pressure (p < 0.001 in both), but no significant differences between contact area values.

### Peak force and pressure location and values

**Fig. 8:**
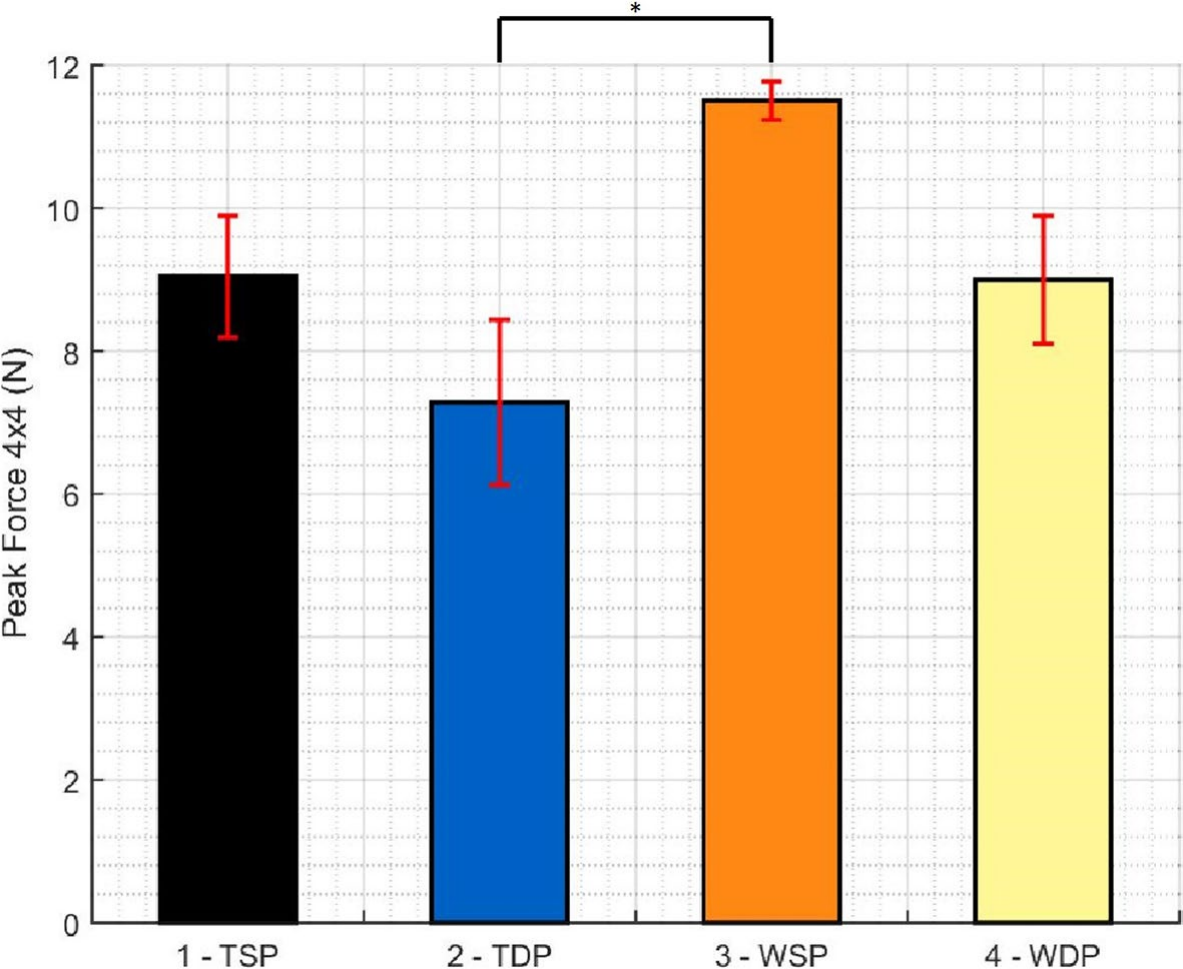
Pairwise comparison of all groups regarding the maximum peak force in a 4 × 4 cells area (25.81mm^2^) (* p < 0.01, ** p < 0.005, *** p < 0.001)

Peak force was located in the posteromedial quadrant in 70% of cases. The highest value was again found in the WSP and the lowest in the TDP group (Fig. 8).

Comparing tapes and wires independently of the type of medial passage, significant higher values of peak force (p = 0.007) and pressure (p = 0.009) occurred in the wire group. Higher values of peak force (p = 0.003) and peak pressure (p = 0.004) were also found in the single passage independent of the type of suture used.

### Force developed by sutures

Higher force was applied by the sutures locked in the PL anchor and in the medial bearing row, independently of the type of material or medial passage (Table 2).

**Table 2:** Descriptive statistics—Mean Force per sensor applied by each suture in each different group

	**TSP**	**TDP**	**WSP**	**WDP**
Mean Force (N)				
PM-PL Suture	.3731	.3529	.5710	.5558
PM-AL Suture	.2826	.1931	.3815	.3496
AM-PL Suture	. 2965	.4283	.4637	.4959
AM AL Suture	.2111	.1930	.2605	.2378
AM-PM line	5.530	5.191	6.871	7.773

When comparing single and double passage repairs no differences was found, but when comparing tapes and wires, the latter generated statistically significant higher force per sensor in all, but in the AM-AL suture (p < 0.001 in PM-PL and AM-PM; p = 0.002 in PM-AL and p = 0.019 AM-PL).

When comparing individual groups, significant statistical differences were only found for the PM-AL suture (Table 3) and for the medial bearing row (Table 4). Again, the highest force was applied by the WSP group, except in the medial region in which WDP surpassed. TDP generated the lowest forces (see Table 2).

**Table 3:** Pairwise comparisons of all groups for mean contact force per sensor applied in the PM-AL suture

Sample 1-Sample 2	Test Statistic	Std. Error	Std. Test Statistic	Sig	Adj. Sig.^a^
TDP-TSP	-6.200	3.742	-1.657	.098	.585
TDP-WDP	-10.000	3.742	-2.673	.008	.045
TDP-WSP	-12.200	3.742	-3.261	.001	.007
TSP-WDP	-3.800	3.742	-1.016	.310	1.000
TSP-WSP	-6.000	3.742	-1.604	.109	.653
WDP-WSP	-2.200	3.742	-.588	.557	1.000

**Table 4:** Pairwise comparisons of all groups for mean contact force per sensor applied by AM-PM line (medial bearing row)

Sample 1-Sample 2	Test Statistic	Std. Error	Std. Test Statistic	Sig	Adj. Sig.^a^
TDP-TSP	-2.400	3.742	-.641	.521	1.000
TDP-WSP	-8.200	3.742	-2.192	.028	.170
TDP-WDP	-13.000	3.742	-3.474	.001	.003
TSP-WSP	-5.800	3.742	-1.550	.121	.727
TSP-WDP	-10.600	3.742	-2.833	.005	.028
WSP-WDP	4.800	3.742	1.283	.200	1.000

### Variation of force in the repair box

**Fig. 9:**
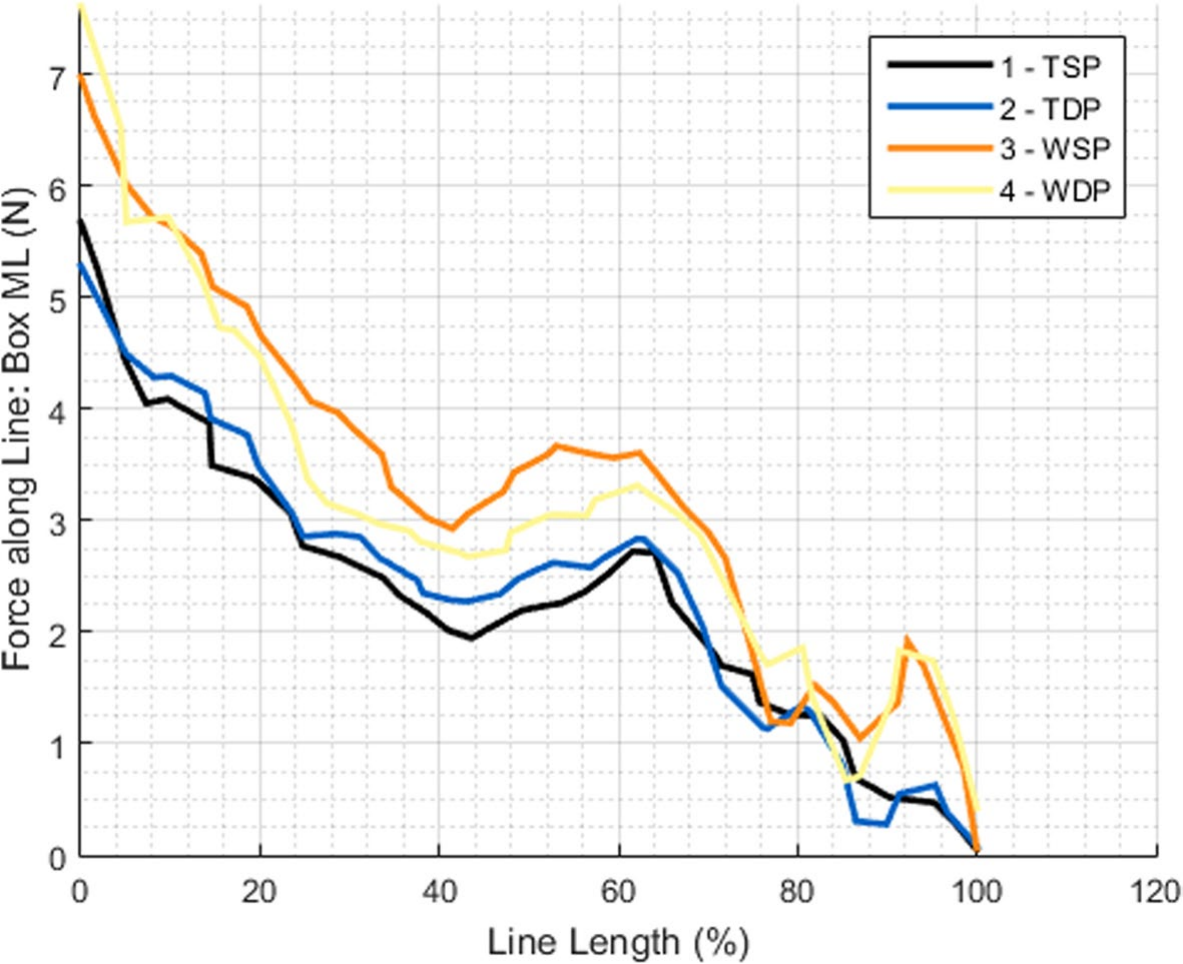
Force variation in the repair box (medial to lateral)

**Fig. 10:**
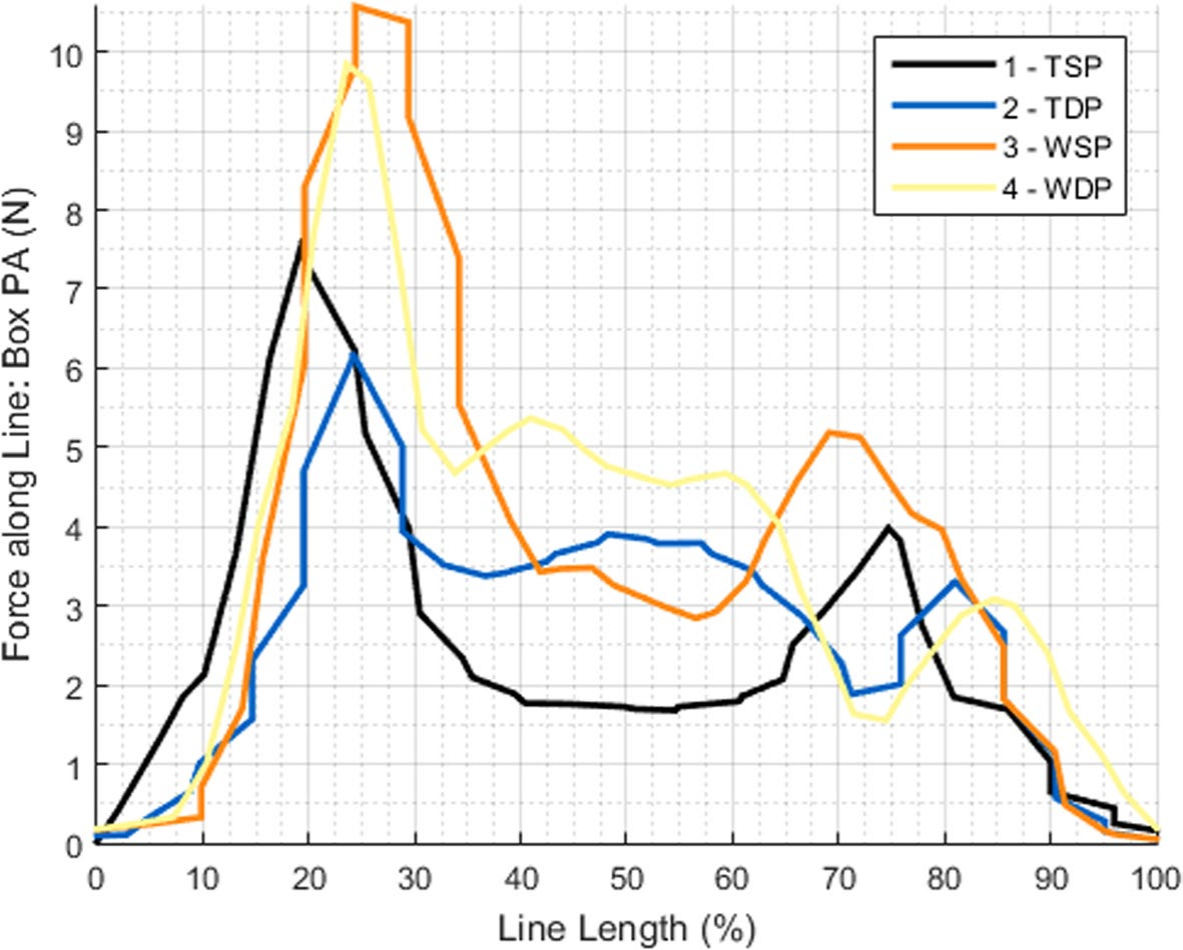
Force variation in the repair box (posterior to anterior)

Figure 9 demonstrates that the force applied in the tendon is maximum in the most medial area of the repair, with higher values for the wire groups, and that it progressively decreases in intensity along the suture path, from medial to lateral. The results also clearly indicate that the posterior half of the repair had the highest contact forces in every test, and again, results were higher for the wire groups (Fig. 10).

## Discussion

A compromise between adequate mechanical stabilization and good biological local environment of the tissues is essential for tendon healing but literature is scarce and unclear regarding the influence of stiffer suture configurations and materials at the TBI. This paper aimed to evaluate the mechanical influence of some surgical options that interfere not only with the mechanical stability of the repair but also with the biological response of the tissues, so that surgeons can better understand the consequences of their individual choices.

The initial hypothesis was partially refused because indeed total contact force and total contact pressure applied are higher when suture wires rather than suture tapes are used, meaning that the compressive effect at the TBI is smaller with tapes. This differs from the results obtained by Huntington [23] and Liu [31] and there may be several reasons for this:

A) We used electronic sensor mapping technology instead of pressure sensitive film [23] or pressure sensitive probes [31]. Other sensors have been previously used in similar settings [8, 34, 42, 48, 50] but the one we used has higher resolution and allows a more precise mapping, especially if compared to the methods used by Liu [31] and Huntington [23].
B) Huntington [23] performed SB repairs with medial anchors that didn´t allow suture slide. According to our data, nonsliding sutures (AM-PL and PM-PL sutures limbs, after AL anchor locking) generate higher contact forces than sutures tensioned at the AL anchor that slid along the AM and PM anchors, possibly explaining the higher values for pressure they obtained, which can be very concerning from a perfusion / tendon vascularization point of view [11, 39].
C) Liu [31] and Huntington [23] used animal models but despite the large sample dimension, specimen variability induces mechanical biases that can obscure final results. This is an important factor to have into account if only mechanical data is being evaluated.
D) Finally, some key experimental variables were not addressed in these reports. As demonstrated by other authors [29, 40, 46], the amount of force applied for lateral suture tensioning has implications in the force and footprint contact pressure, which means that in order to ensure trial homogeneity and study reproducibility, suture tension control in the lateral row is mandatory and to our best knowledge this wasn´t performed.

Despite the differences shown above regarding total contact force and pressure, suture tapes and wires didn´t generate significant different total contact area, which is in accordance with Huntington´s paper [23]. This means that under the same bone and tendon conditions, when controlled lateral suture tension is used, tapes compared with suture wires, generate similar tendon-bone contact area and lower contact force and pressure, in opposition to what had been previously described [23, 31]. This theoretical mechanical disadvantage can reveal itself beneficial and explain the superior clinical results obtained by slightly less stable and stiff repairs [27, 32, 33, 38, 47], when compared to those that the literature demonstrated to be the most biomechanically stable ones, namely those with smaller gap formation [1, 33], higher contact pressure (especially in the medial bearing row) [27, 42], contact area [27, 42], stiffness [33] and resistance to failure [1, 32].

Our work also confirmed that, not only does the total area of contact increase with the use of individually passed sutures limbs (double passage) in the medial cuff, but also this technical variation tendentially decreases the total force and total pressure applied at the TBI. When compared to single passage, double medial passage led to a total contact force decrease of 3,1% if tapes were used and 5,6% if wires were chosen. Also, total contact pressure decreased from 6,5% in tapes group and 12,2% in case of the wires group. This data seems especially relevant because the distance between the most anterior and posterior passage sites was similar in single and double passage repairs, so even if the tendon “Repair Box” is similar, higher number of suture passages points medially, increases the total contact area between tendon and bone, while promoting a minor decrease in total contact force and pressure, eventually favoring tendon perfusion and tendon healing, while allowing better tension stress distribution over the tendon once healing has occurred.

It was also demonstrated that the use of double passage lowered peak force and pressure at the most compressed areas, which can also lower the risk of biological failure in those specific locations [26].

To our knowledge this is the first report demonstrating the influence of multiple passage points in total contact force, total contact pressure, total contact area and peak force and pressure at the TBI.

The type of knotless repair tested also provides insight on the mechanical consequences of medial anchors with locked sutures versus medial anchors with sliding sutures, especially regarding contact force pattern.

In this experimental setup, both medial anchors allowed suture sliding, so when the first lateral anchor was placed (AL) and one suture limb of each medial anchors pulled (AM-AL and PM-AL sutures), sliding naturally occurred and at lower tension values for wires when compared to tapes, in line with Leishman [30] report (wires slid at an interval between 25 and 50 N and tapes slid between 50-75 N (no exact value was obtained because this type of tensioner doesn´t allow sequential numeric tension measurement)). However, after AL anchor locking, suture limb pulling on the PL anchor (AM-PL and PM-PL sutures) did not show suture sliding, so consistent and reproducible 75 N lateral suture tensioning was possible, with a clearly higher compressive effect at the posterior portion of the “Repair Box”, stabilized by the “non-sliding” AM-PL and PM-PL sutures, when compared with the anterior area that had been stabilized by the AM-AL and PM-AL sutures (Fig. 10).

This corroborates the findings of Park [40] that stated the importance of controlling lateral tension, not only in biomechanical studies but also in the clinical setting as higher lateral tension translates into greater force application at the tendon, moreover if tied TOE repairs or full medial locked knotless TOE repairs are chosen, because continuous lateral tension in non-sliding sutures can promote growing and potentially supraphysiological compression force at the TBI with detrimental mechanical and tendon perfusion consequences [39], especially if wires and single medial suture passage are used.

In fact, most of our findings help to support some of other authors´ hypothesis [10, 54] in which tension overload of the suture-tendon interface at the medial bearing row, over-tensioning of the medial repair, over-medialization of suture passage, creation of large holes in the rotator cuff (by instruments or eventually by a larger number of sutures in the same hole) [45], increased abrasion induced by high resistance sutures [15] and suture induced tendon necrosis [26, 39], were possible causes for type 2 retears.

The evaluation of the mean force applied at the path of sutures and in the medial row also confirmed the previous global overview, in which wires create higher contact force especially in the posterior sutures and in the medial bearing row. Also, and as expected, contact force in the repair box tends to be higher in the most medial region and lowers progressively as we approach the lateral side of the repair.

Both tape and wire results demonstrated higher medial bearing row contact force and pressure meaning that the medial row is the area subjected to the highest tensional stress.

Considering McCarron [35] demonstration that, even if healed to the bone, all tendons tend to retract after surgical repair, and also taking into account the obtained data, Trantalis´s [54] hypothesis seems plausible because excessive force applied in the medial bearing row not only creates a local area of stress concentration as described by Park [42], but also stress shields the lateral tendon from self-reinforcement. Aggravated by local tendon hypoperfusion [26], normal tendon retraction can´t occur, which can increase the risk for type 2 retears, and in light of our results, this is probably favored by wire use and excessive tension in the lateral sutures [29].

This paper has some strong features that should be considered such as the use of a mechanical model that, despite precluding immediate clinical translation allows for a more reproducible evaluation of mechanical data, without the biological variability induced by biological specimens.

Also, the use of a template and a single sized needle for suture passage contributed to a reproducible application of anchors and sutures and trial homogeneity.

The higher resolution of this specific sensor when compared to others previously reported [8, 34, 42, 48, 50] is also a strong feature that may have allowed a more reliable measurement of force and pressure mapping, without the need for sensor penetration/damage to prevent dislocation, following manufacturer instructions.

At last, and to our best knowledge, this is the first report that not only compares suture tapes and wires in a simulated rotator cuff repair using controlled lateral tension but also evaluates the influence of medial suture passage pattern in contact force, pressure and area. A specific 75 N of lateral row tension was used based in the previous reports of Park [40] showing that beyond 90 N of lateral tension, tendon to bone contact area did not increase, so according to the type of tensioners used, 75 N appeared the best option.

There are also some methodological limitations that should be highlighted. First, due to its dimension, this specific sensor had to be folded to fit the mock repair, but the sensors´ integrity was respected, and this was confirmed upon calibration.

It is also impossible to assure that similar results could be achieved if the sensor had been perfectly adjusted to the mechanical model, but the calibration performed before the experimental trials and previous validation studies performed in similar sensors [34] validates the data obtained.

Also, the low number of essays per group can limit the robustness of our results. This was due to the costs involved, especially anchor wise. Despite this, several other reports have used an approximated number of trials while using animal or cadaver models, which have a higher variability in terms of bone and tendon mechanical properties [6, 7, 22, 31, 46, 48].

Another specific limitation is related to suture passage path location in the sensor, which was inferred considering sensor and software obtained data and also the distance between suture holes and the force pattern in the repair box. Although subjected to variability, the same author performed all the observations and measurements.

**Fig. 11:**
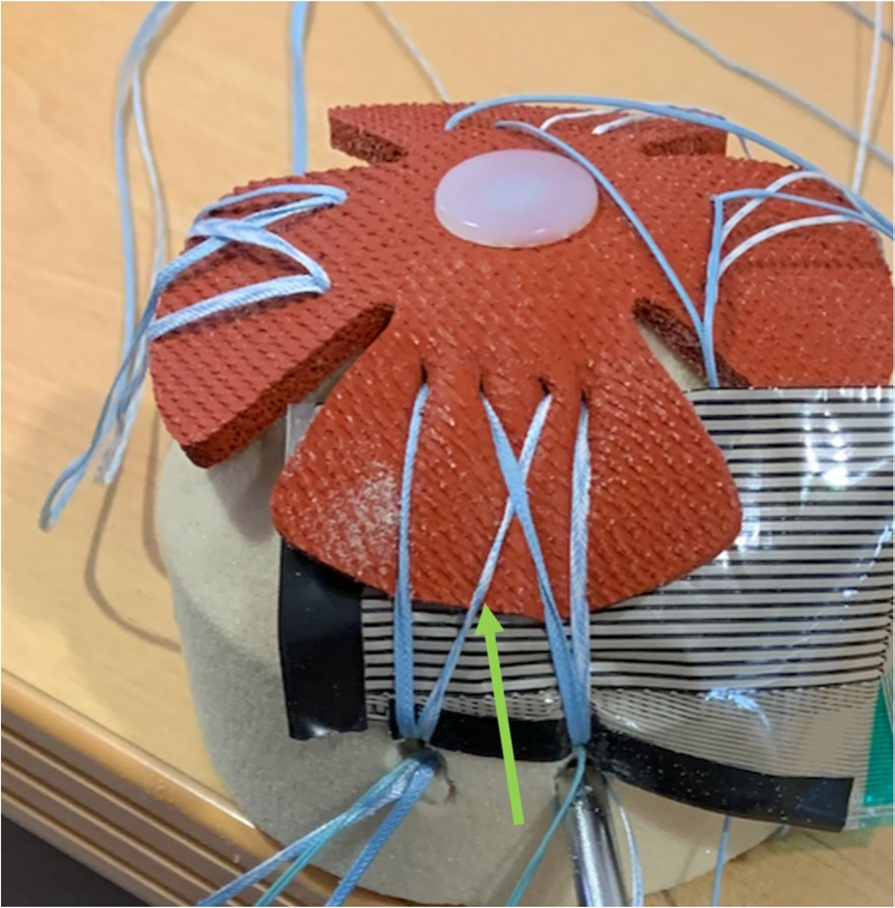
Suture tape in a TDP trial macroscopically behaving as a wire (see PM-AL suture—green arrow)

At last, the specific tapes used in this paper do not have a core so these can behave like a wire in some assemblies (see Fig. 11), something that also happens in the clinical setting but in this case, it can create a confounding factor when evaluating tape results.

## Conclusion

The use of tapes decreases total contact force, total contact pressure, peak force and pressure at the tendon-bone interface, and double (isolated) suture limb medial passage also decreases those parameters, while increasing contact area. These results offer a better understanding of the mechanical interactions at the tendon-bone interface when using different suture materials and repair configurations and open the door for some technical adaptations that can improve surgical outcomes.

## Supplementary Information

**Additional file 1.**

## Data Availability

Not applicable.

## Availability of data and materials

See supplementary material. All remaining data will be provided upon request to the corresponding author.
