# Supplementary material for: Why are tapes better than wires in knotless rotator cuff repairs? An evaluation of force, pressure and contact area in a tendon bone unit mechanical model
Source: J Exp Orthop. 2021 Feb 3;8:9. doi: 10.1186/s40634-020-00321-y (PMC7859138; doi:10.1186/s40634-020-00321-y)
Supplement: Supplementary file 1 — Additional file 1. [file 40634_2020_321_MOESM1_ESM.docx]

**Table 2- Descriptive Statistics (Peak force and pressure)**

|  | | **TSP** | **TDP** | **WSP** | **WDP** |
| --- | --- | --- | --- | --- | --- |
| **Peak values in 4x4 cells** | Force (N) | 9.04 | 7.28 | 11.50 | 9.00 |
|  | Pressure (MPa) | .3522 | .2823 | .4458 | .3488 |
